# Extracts and Constituents of *Rubus chingii* with 1,1-Diphenyl-2-picrylhydrazyl (DPPH) Free Radical Scavenging Activity

**DOI:** 10.3390/ijms12063941

**Published:** 2011-06-14

**Authors:** Hsiou-Yu Ding

**Affiliations:** Institute of Cosmetics Science, Chia Nan University of Pharmacy and Science, 60 sec. 1 Erh-Jen RD, Jen-Te, Tainan, 71710, Taiwan; E-Mail: hsiou221@yahoo.com.tw; Tel.: +886-6-2664911; Fax: +886-6-2670324

**Keywords:** antioxidant, *Rubus chingii*, 1,1-diphenyl-2-picrylhydrazyl (DPPH), rubusine

## Abstract

The 1,1-diphenyl-2-picrylhydrazyl (DPPH) free radical scavenging activity of the fruits of *Rubus chingii* was studied *in vitro*. Ethanolic extract, ethyl acetate and *n*-butanol fractions from dried *R. chingii* fruits revealed strong DPPH free radical scavenging activity with IC_50_ values of 17.9, 3.4 and 4.0 μg/mL, respectively. The ethyl acetate and *n*-butanol fractions were further purified by a combination of silica gel chromatography, Lobar RP-8 chromatography, and high-pressure liquid chromatography (HPLC). Nine compounds were isolated, where methyl (3-hydroxy-2-oxo-2,3-dihydroindol-3-yl)-acetate (**2**), vanillic acid (**5**), kaempferol (**7**), and tiliroside (**9**) showed stronger DPPH free radical scavenging activity than that of ascorbic acid (131.8 μM) with IC_50_ values of 45.2, 34.9, 78.5, and 13.7 μM, respectively. In addition, rubusine (**1**) is a new compound discovered in the present study and methyl (3-hydroxy-2-oxo-2,3-dihydroindol-3-yl)-acetate (**2**), methyl dioxindole-3-acetate (**3**), and 2-oxo-1,2-dihydroquinoline-4-carboxylic acid (**4**) were isolated from the fruits for the first time.

## 1. Introduction

Fruits and vegetables are known to contain different antioxidant compounds, and high consumption of these products has long been associated with a lower incidence of degenerative diseases. This beneficial association is considered to be partially due to the various antioxidant compounds present in these foods; these antioxidants scavenge free radicals and, thereby, reduce the manifestation of degenerative pathologies [1–3]. Colorful fruits and green leafy vegetables are rich sources of phenolic and flavonoid compounds, which exhibit a range of antioxidant, antibacterial, anti-inflammatory, antiallergic, hepatoprotective, vasodilatory, and neuroprotective activities [4–7].

For many years, dried fruits of *R. chingii* Hu (Rosaceae), referred to as “fu-pen-zi” in Chinese, have been used as a food and a tonic in traditional Chinese medicine [8]. Fupenzi is used to improve the functioning of the kidney and to treat seminal discharge and excessive polyuria [9]. In recent decades, modern pharmacological experiments have revealed that *R. chingii* has immunomodulatory effects [10] on bacterial infection, anxiety, pain, inflammation [11], and the hypothalamus-pituitary-sex gland axis [12]. Previous phytochemical studies of this plant have led to the isolation of triterpene acids, flavonoids, phenolics, and steroids [13–18]. In the present study, the DPPH free radical scavenging activity of the extracts and constituents of Chinese medicine was investigated.

## 2. Results and Discussion

### 2.1. Isolation and Identification of Alkaloids, Phenolics, and Flavonoids from *R. chingii*

**Figure 1:**
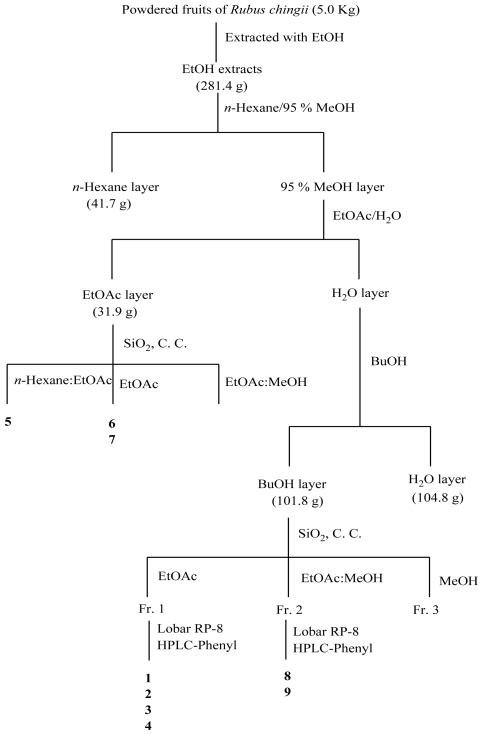
Isolation scheme for *R. chingii*.

**Figure 2:**
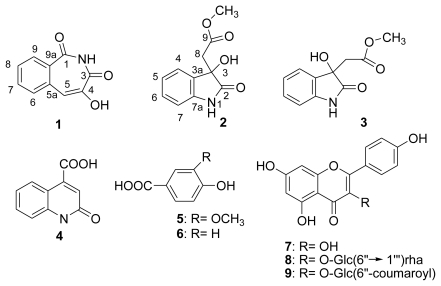
Chemical structures of compounds 1–9.

The ethanolic extract of the dried fruits of *R. chingii* was partitioned into *n*-hexane, ethyl acetate, *n*-butanol, and water fractions. The ethyl acetate and *n*-butanol fractions were subjected to silica gel chromatography, prep-Lobar RP-8, and preparative high-pressure liquid chromatography (prep-HPLC) to isolate one new alkaloid compound, rubusine (**1**), together with eight known compounds (Figure 1). Their structures were established on the basis of spectral evidence (Figure 2). The compounds, which comprised three alkaloids: methyl (3-hydroxy-2-oxo-2,3-dihydroindol-3-yl)-acetate (**2**) [19], methyl dioxindole-3-acetate (**3**) [20], 2-oxo-1,2-dihydroquinoline-4-carboxylic acid (**4**) [21]; two phenolics: vanillic acid (**5**) [18] and *p*-hydroxybenzoic acid (**6**); three flavonoids—kaempferol (**7**) [15], nicotiflorin (**8**) [17], and tiliroside (**9**) [14] were identified by comparing the spectroscopic data with those in reported literature.

Rubusine (**1**) was an amorphous powder in methanol. HR-EIMS revealed an [M^+^] at m/z 189.0420, corresponding to the molecular formula C_10_H_7_NO_3_. The IR spectrum of **1** revealed absorption bands at ν_max_ 3450–3200 (OH), 2978, 2895 (NH), and 1650 (C = O) cm^−1^ indicating hydroxyl, imide, and ketone groups, respectively. The ^1^H NMR spectrum of **1** exhibited an aromatic ring at *δ* 7.22 (t, *J* = 7.7 Hz), 7.36 (d, *J* = 7.7 Hz), 7.54 (t, *J* = 7.7 Hz), and 8.13 (d, *J* = 7.7 Hz). The ^13^C NMR spectrum was *δ* 115.8, 122.3, 126.1, 130.9, 139.4, and 141.2. The ^13^C NMR chemical shifts for two ketone groups at *δ* 161.1 were in the B ring. The B ring has a double bond as indicated by the ^1^H NMR peak of *δ* 6.86. The ^13^C NMR peaks of *δ* 123.4 and 166.8 suggest that the double bond possesses one hydroxyl group. The results of correlation spectroscopy (COSY), heteronuclear multiple quantum coherence (HMQC), heteronuclear multiple bond connectivity (HMBC), and NOESY of the ^1^H and ^13^C NMR signals are shown in Table 1. These data agreed with the structure of rubusine (**1**) (Figure 2).

Methyl (3-hydroxy-2-oxo-2,3-dihydroindol-3-yl)-acetate (**2**) was a pale tinted powder and exhibited [*α*]_D_ +10° (MeOH). HR-EIMS revealed an [M^+^] at *m/z* 221.0683, corresponding to the molecular formula C_10_H_7_NO_3_. The UV, IR, ^1^H and ^13^C NMR spectral data of **2** were similar to those of methyl dioxindole-3-acetate (**3**), except that the methylene proton peaks were recorded at *δ* 3.05 and 3.08 (each d, *J* = 15.6 Hz, H-8), and shifted downfield. In addition, C-2 (*δ* 180.9) and C-7a (*δ* 143.7) were shifted downfield by *δ* 6.1 and *δ* 5.3, respectively. Comparing with the ^1^H and ^13^C NMR data of compound **3**, indicated that compound **2** may be an enantiomer of compound **3**. The ^1^H NMR spectrum of **2** also exhibited an ABCD system of aromatic rings at *δ* 6.88 (dd, *J* = 7.5, 1.0 Hz), 7.01 (td, *J* = 7.5, 1.0 Hz), 7.25 (td, *J* = 7.5, 1.0 Hz), and 7.35 (dd, *J* = 7.5, 1.0 Hz) and a methoxyl group of esters at *δ* 3.46. HMBC spectrum of compound **2** showed H-8 (*δ* 3.05 and 3.08; each d, *J* = 15.6 Hz) correlation to C-2 (*δ* 180.9), C-3 (*δ* 42.3), C-3a (*δ* 131.8), and C-9 (*δ* 171.1). H-4 (*δ* 7.35, dd, *J* = 7.5, 1.0 Hz) was correlated with C-3 (*δ* 42.3), C-3a (*δ* 131.8), and C-7a (*δ* 143.7). Taken together, the structural data point to **2** being methyl (3-hydroxy-2-oxo-2,3-dihydroindol-3-yl)-acetate (**2**). The ^1^H- and ^13^C-NMR signals of **2** were completely assigned using contemporary 2-D NMR techniques (COSY-45, HMQC, HMBC and NOESY) and are listed in Table 2.

### 2.2. DPPH Free Radical Scavenging Activity of Extract, Partitions, and Compounds of *R. chingii*

Antioxidants are extensively used in cosmetics, food, and medicine because they can counteract cellular free radicals and reduce metal ions, thereby, interrupting the oxidizing chain reaction before any damage occurs. Antioxidants, therefore, can combat many health problems [22]. The DPPH free radical scavenging activity of the fupenzi fruit extracts and partitions were in the order: ethyl acetate layer, *n*-butanol layer, water layer, ethanol extract, ascorbic acid, *n*-hexane layer, and ascorbic acid (Table 3). The compounds methyl (3-hydroxy-2-oxo-2,3-dihydroindol-3-yl)-acetate (**2**), vanillic acid (**5**), kaempferol (**7**), and tiliroside (**9**) showed significant free radical scavenging activity with IC_50_ values of 45.2, 34.9, 78.5, and 13.7 μM, respectively. Compound **9** appeared to 9.62-fold as potent as ascorbic acid, whereas **2**, **5**, and **7** were about 2.92, 3.78, and 1.68-fold, respectively, as potent. Compound **8** had the same antioxidant activity as that of ascorbic acid (Table 4).

In the present study, the radical scavenging activity of the compounds tested was influenced by the number and the location of the hydroxyl groups, glycosylation, and other substitutions [23]. Compared with nicotiflorin (**8**, IC_50_ = 130.6 μM, 23), the DPPH scavenging activity of kaempferol (**7**, IC_50_ = 78.5 μM) was 1.66-fold higher. However, tiliroside (**9**, IC_50_ = 13.7 μM) exhibited 5.73-fold higher activity than that of kaempferol (**7**). Many of the benefits associated with consumption of phenolic-rich foods are correlated with their antioxidant activities [24]. Research has suggested that phenol may prevent lipid peroxidation via hydrogen atom donation from the hydroxyl group attached to the benzene ring. The present study further demonstrated that the phenolic compounds possessing hydroxyl groups exhibited much higher DPPH free radical scavenging activity. Vanillic acid (**5**, IC_50_ = 34.9 μM) exhibited higher activity when compared with *p*-hydroxybenzoic acid (**6**, IC_50_ = 311.5 μM) [25]. An adjacent substituted methoxyl group in the aromatic ring was the most significant difference between the two phenolics. A possible explanation for the difference in DPPH free radical scavenging activity is the difficulty in forming the reaction complex between the phenolics and the free radicals. The alkaloid compound 2-oxo-1, 2-dihydroquinoline-4-carboxyloic acid (**4**, IC_50_ = 1216.1 μM) has no hydroxyl group, and its DPPH free radical scavenging activity was not significant.

## 3. Experimental Section

### 3.1. Plant Material

The dried fruits of *R. chingii* were purchased from Chung-Yuan Company, Kaoshiung, Taiwan. Prof. Hang Chang Lin at the National Defense Medical Center, Taipei, Taiwan, identified the plant. The *R. chingii* specimen (No. 960801) has been deposited at the herbarium of the Chia Nan University of Pharmacy and Science, Tainan, Taiwan.

### 3.2. General Experimental Procedures and Apparatus

We used a Yanagimoto micromelting point apparatus to determine the melting points, which are reported as uncorrected values. Optical rotations were measured in a GENESYS 20 polarimeter (Rochester, New York, NY, USA) The IR spectra were recorded on KBr disks with a Perkin-Elmer 983 G spectrophotometer, and the UV spectra were obtained on a Shimadzu UV-160 spectrometer. ^1^H and ^13^C NMR spectra were determined with a Bruker AM-500 spectrometer using DMSO-d_6_ and MeOH-d_4_. The FABMS measurements were measured using a JEOL JMX-HX110 mass spectrometer. The EIMS were recorded at 70 eV using a Finnigan MAT TSQ 46C GC/MS/MS/D spectrometer.

The column chromatography was carried out on silica gel (70–230 mesh, Merck). The preparative liquid chromatography was performed by a Shimadzu LC-8A chromatograph on a reverse C-18 column (Nacalai Teaqe shim-pack, pre-CODSL, 15 um, 50 × 250 mm, flow rate 15 mL/min). Two Shimadzu LC-8A pumps and a Shimadzu SCL-8A system controller were used for preparative HPLC (Shimadu, Tokyo, Japan). The chromatography peaks were detected by UV (254 nm) using a Shimadzu SPD6VA UV detector. A FMI RP-SY-ICSC Lobar pump was used for the low pressure liquid chromatography (Lobar). Silica gel 60 (Merck 70–230 mesh, 230–400 mesh; ASTM) was used for the column chromatography, and silica gel 60 F_254_ (Merck, Darmstadt, Germany) was used for the thin-layer chromatography.

### 3.3. Extraction, Partition, and Isolation of R. chingii Compounds

The detailed purification procedure involved in the extraction and separation of the nine compounds from *R. chingii* is shown in Figure 1. The dried fruits (5.0 kg) were ground in a power grinder and soaked in 95% ethanol (×4) at room temperature for 3 days. The extracts were decanted, filtered under vacuum, and concentrated in a rotating evaporator to produce dark brown syrup (extraction yield 5.63% (w/w) dry weight) of 281.4 g. The crude extract was partitioned between *n*-hexane and 95% methanol. The 95% methanol fraction was concentrated and partitioned between ethyl acetate and water. The aqueous extract was again partitioned between *n*-butanol and water. The resulting portion generated fractions in the *n*-hexane layer (41.7 g, 14.8% (w/w) yield), ethyl acetate layer (31.9 g, 11.3% (w/w) yield), *n*-butanol layer (101.8 g, 36.2% (w/w) yield), and H_2_O layer (104.8 g, 37.2% (w/w) yield). The ethyl acetate layer was introduced into the silica gel chromatography column and eluted with an *n*-hexane–ethyl acetate mix (3:1, 1:1), an ethyl acetate–ethyl acetate-methanol mix (3:1, 1:9), and methanol to obtain vanillic acid (**5**, 8.0 mg), *p*-hydroxybenzoic acid (**6**, 67.0 mg), and kaempferol (**7**, 6.5 mg). The *n*-butanol layer was introduced to the chromatography column on silica gel with ethyl acetate, the ethyl acetate–methanol mix (19:1, 9:1, 4:1, 1:1), and methanol. Re-chromatography of the first fraction on prep-Lobar RP-8 was conducted, followed by HPLC separation, which yielded rubusine (**1**, 56.0 mg), methyl (3-hydroxy-2-oxo-2,3-dihydroindol-3-yl)-acetate (**2**, 71.0 mg), methyl dioxindole-3-acetate (**3**, 22.0 mg), and 2-oxo-1,2-dihydroquinoline-4-carboxylic acid (**4**, 16.0 mg). Similarly, the second fraction produced tiliroside (**9**, 16.0 mg), and the third fraction produced nicotiflorin (**8**, 60.0 mg).

Rubusine (**1**): Colorless powder; mp 291–292 °C; UV λ_max_ nm: 210.0, 230.0, 278.0, 289.0; IR ν_max_ cm^−1^: 3450–3200, 2978, 2895, 1650; HREI-MS: The calculated molecular mass of C_10_H_7_NO_3_, m/z: 189.0426 [M]^+^, Measured: 189.0420; EI-MS m/z (posit., rel. int. %): 189 ([M]^+^, 100), 161 (12), 144 (31), 117 (34); ^1^H and ^13^C NMR data are presented in Table 1.

Methyl (3-hydroxy-2-oxo-2,3-dihydroindol-3-yl)-acetate (**2**): white powder; mp 131–132 °C; [*α*]_D_ +10° (*c* = 1.0, MeOH); UV λ_max_ nm: 254.3, 285.5; IR ν_max_ cm^−1^: 3550–3280, 2982, 2894, 1646; HREIMS *m/z* 221.0688 [M]^+^ (calculated for C_11_H_11_NO_4_, 221.0683); EI-MS m/z (posit., rel. int. %): 221 ([M]^+^, 48), 161 (81), 148 (100), 133 (20), 120 (38); ^1^H and ^13^C NMR data are presented in Table 2.

Methyl dioxindole-3-acetate (**3**): Pale yellow oil; [*α*]_D_ −3.1°(*c* = 1.0, MeOH); UV λ_max_ (MeOH) nm: 252.8; IR *v*_max_ (KBr) cm^−1^: 3590–3125, 2975, 1649; EI-MS m/z (posit., rel. int. %): 221 ([M]^+^, 22), 204 (100), 162 (90); ^1^H-NMR(CD_3_OD, 500 MHz): *δ* 2.81, 3.07 (each 1H, d, *J* = 16.0 Hz, H-8), 3.73 (3H, s, OCH_3_), 6.92 (1H, dd, *J* = 7.8, 1.0 Hz, H-4), 7.05 (1H, td, *J* = 7 .8, 1.0 Hz, H-6), 7.28 (1H, dd, *J* = 7.8, 1.0 Hz, H-5),7.37(1H, dd, *J* = 7.8, 1.0 Hz, H-7); ^13^C NMR (CD_3_OD, 125 MHz): *δ* 43.1 (C-8), 53.5 (OCH3), 75.2 (C-3), 117.2 (C-7), 124.5 (C-5), 125.9 (C-6), 127.3 (C-4), 131.1 (C-3a), 138.4 (C-7a), 170.8 (C-9), 174.8 (C-2).

### 3.4. DPPH (1,1-Diphenyl-2-picryhydrazyl) Free Radical Scavenging Assay

To measure the antioxidant activity, the 1,1-diphenyl-2-picrylhydrazyl (DPPH) free radical scavenging activity was determined by the spectro-photometric method previously described [26]. A methanol solution of a sample (100 μL) was mixed with a 0.5 mM DPPH methanol solution (800 μL) and 0.1 M acetate buffer (pH 5.5; 100 μL). The absorbance of the mixture at 517 nm was measured after standing for 30 min. The IC_50_ value was determined as the concentration of each sample required to give 50% of the absorbance shown by the blank test.

## 4. Conclusions

This study demonstrated that the ethanolic extract, ethyl acetate, *n*-butanol, and water fractions of fupenzi exhibit potent DPPH free radical scavenging activity. Nine compounds were isolated, including rubusine (**1**), methyl (3-hydroxy-2-oxo-2,3-dihydroindol-3-yl)-acetate (**2**), methyl dioxindole-3-acetate (**3**), 2-oxo-1,2-dihydroquinoline-4-carboxylic acid (**4**), vanillic acid (**5**), *p*-hydroxybenzoic acid (**6**), kaempferol (**7**), nicotiflorin (**8**), and tiliroside (**9**). Compound **1** is a new alkaloid compound. Compounds **2**, **5**, **7**, **8**, and **9** showed potent DPPH free radical scavenging activity. These results suggest that fupenzi may be used as a natural antioxidant to improve the quality, stability, and safety of cosmetics, foods, and medicines.

## Figures and Tables

**Table 1:** NMR spectroscopic data (500 MHz, DMSO-*d*_6_) of rubusine (**1**).

C	*δ*_C_	*δ*_H_ (*J* in Hz)	HMBC	NOESY	COSY
1	161.4				
3	161.4		H5		
4	166.8		H5		
5	123.4	6.86, s	H6		
5a	139.4		H7,8,9		
6	115.8	7.36 d (7.7)	H5,7,8	H6/H7	H6/H7
7	130.9	7.54 t (7.7)	H6,8,9	H7/H6,8	H7/H6,8
8	122.3	7.22 t (7.7)	H6	H8/7,9	H8/7,9
9	126.1	8.13 d (7.7)	H7,8	H9/H8	H9/H8
9a	141.2		H5,6,9		

**Table 2:** NMR spectroscopic data (500 MHz, CD_3_OD) of methyl (3-hydroxy-2-oxo-2,3- dihydroindol-3-yl)-acetate (2).

No.	*δ*_C_	*δ*_H_ (*J* in Hz)	HMBC	NOESY	COSY
2	180.9 s				
3	74.9 s				
3a	131.8 s				
4	125.3 d	7.35 dd (7.5, 1.0)	3, 6, 7, 7a	H5	H5
5	123.7 d	7.01 td (7.5, 1.0)	3, 3a, 4, 6, 7, 7a	H5/H4,6,8	H5/H4,6
6	131.1 d	7.25 td (7.5, 1.0)	3a, 4, 5, 7, 7a	H6/H5,7	H6/H5,7
7	111.4 d	6.88 dd (7.5, 1.0)	3, 3a, 4, 5, 7a	H7/H6	H7/H6
7a	143.7 s				
8	42.3 t	3.05 d (15.6)	2, 3, 3a, 9	H8/H5,OCH	
		3.08 d (15.6)	2, 3, 3a, 9	CH_3_	
9	171.1 s				
OCH_3_	52.2 q	3.46 s	9	OCH_3_/H8	

**Table 3:** DPPH radical scavenging activity of the ethanolic extract and partitioned layers.

	IC_50_ (μg/mL)	Yield (%)
EtOH ext.	17.9 ± 0.50	5.63
*n*-hexane	62.2 ± 0.38	14.8
Ethyl acetate	3.4 ± 0.43	11.3
*n*-butanol	4.0 ± 0.46	36.2
Water	4.7 ± 0.45	37.2
Ascorbic acid^a^	23.2 ± 0.65	—

a Compound as positive control.

**Table 4:** IC_50_ values of the constituents isolated from the fruits of *R. chingii* against DPPH free radical.

Compounds	IC_50_ (μM)
Rubusine (**1**)	431.5 ± 2.77
Methyl(3-hydroxy-2-oxo-2,3-dihydroindol-3-yl)-acetate (**2**)	45.2 ± 0.87
Methyl dioxindole-3-acetate (**3**)	294.1 ± 2.33
2-Oxo-1,2-dihydroquinoline-4-carboxylic acid (**4**)	1216.9 ± 4.52
Vanillic acid (**5**)	34.9 ± 1.01
*p*-hydroxybenzoic acid (**6**)	311.5 ± 0.60
Kaempferol (7)	78.5 ± 1.53
Nicotiflorin (**8**)	130.6 ± 1.33
Tiliroside (**9**)	13.7 ± 0.48
Ascorbic acid^a^	131.8 ± 0.65

Each value represents the mean SD (*n* = 3);

a Compound as positive control.
